# The regulatory effect of hyaluronan on human mesenchymal stem cells’ fate modulates their interaction with cancer cells in vitro

**DOI:** 10.1038/s41598-021-00754-0

**Published:** 2021-10-27

**Authors:** Christian Vogeley, Özer Degistirici, Sören Twarock, Jessica Wladarz, Oliver Reiners, Tobias Gorges, Jens W. Fischer, Roland Meisel, Katharina Gorges

**Affiliations:** 1 Institute for Pharmacology and Clinical Pharmacology, University Hospital of the Heinrich-Heine-University, Düsseldorf, Germany; 2 Division of Pediatric Stem Cell Therapy, Department of Pediatric Oncology, Hematology and Clinical Immunology, Center for Child and Adolescent Health, Medical Faculty, Heinrich-Heine University, Düsseldorf, Germany; 3 Department of Tumor Biology, University Medical Center, Hamburg-Eppendorf, Hamburg, Germany

**Keywords:** Breast cancer, Cancer microenvironment, Metastasis, Mesenchymal stem cells, Stem-cell differentiation

## Abstract

Metastatic spread of cancer cells into a pre-metastatic niche is highly dependent on a supporting microenvironment. Human bone marrow-derived mesenchymal stem cells (bmMSCs) contribute to the tumor microenvironment and promote cancer metastasis by inducing epithelial-to-mesenchymal transition and immune evasion. The underlying mechanisms, however, are incompletely understood. The glycosaminoglycan hyaluronan (HA) is a central component of the extracellular matrix and has been shown to harbor pro-metastatic properties. In this study we investigated the highly disseminating breast cancer and glioblastoma multiforme cell lines MDA-MB-321 and U87-MG which strongly differ in their metastatic potential to evaluate the impact of HA on tumor promoting features of bmMSC and their interaction with tumor cells. We show that adipogenic differentiation of bmMSC is regulated by the HA-matrix. This study reveals that MDA-MB-231 cells inhibit this process by the induction of HA-synthesis in bmMSCs and thus preserve the pro-tumorigenic properties of bmMSC. Furthermore, we show that adhesion of MDA-MB-231 cells to bmMSC is facilitated by the tumor cell-induced HA-rich matrix and is mediated by the HA-receptor LAYN. We postulate that invasive breast cancer cells modulate the HA-matrix of bmMSC to adapt the pre-metastatic niche. Thus, the HA-matrix provides a potential novel therapeutic target to prevent cancer metastasis.

## Introduction

Metastatic spread of tumor cells is a pivotal process of cancer progression and a critical prognostic factor for disease burden and patient survival. The stages and premises of tumor cell dissemination and metastasis formation have been intensely studied since Paget put forward the seed-and-soil theory almost a century ago^1^. The cellular and structural constituents of the bone marrow (BM) represent the soil for a variety of cancer entities including gastrointestinal cancer, breast cancer and glioblastoma^2–4^. Interestingly, the endurance and behavior of disseminated tumor cells in the BM is highly variable. Disseminated breast cancer cells harbor the potential to become dormant while residing in the BM niche but, when reactivated, may give rise to metastatic disease even many years after the resection of the primary tumor^5^. In contrast, even though hematogenous spread is an intrinsic feature of glioblastoma multiforme, bone marrow metastasis is exceedingly rare. Immunocompromised patients, however, reveal a higher frequency of BM metastatic disease indicating that disseminated tumor cells are rapidly cleared from the BM in an immunocompetent host. The reasons for the differences in the metastatic potential between these and other tumor entities remain largely unknown. Apart from tumor cell-intrinsic mechanisms, it has been shown that the tumor cell behavior in the metastatic niche is highly dependent on non-cell autonomous interactions within the microenvironment. Unravelling the differences in intercellular communications between these tumor types holds great promise for the identification of novel therapeutic approaches.

Several cell types which have been implicated in developing a hospitable metastatic niche in the bone marrow are derived from mesenchymal stem cells (MSC)^6^. MSC themselves present only a small cellular fraction of the BM but are essential for bone homeostasis, participate in the processes of connective tissue regeneration^7, 8^ and exhibit pleiotropic immunomodulatory functions^9, 10^, e.g. by inhibition of T-cell responses^11^. Tumor-associated MSCs have been shown to constantly remodel the tumor microenvironment and thus support tumor progression. Amongst other things MSC enhanced tumor growth^12^ by paracrine secretion of NRG1/HER3^13^ and by exosome shuttled microRNAs^5^. Given the plethora of studies showing the tumor promoting effect of MSCs, it is likely that in a reversing circuit disseminated tumor cells also shape the cellular landscape of the BM microenvironment. Supporting evidence is provided by the finding that after chemotherapy a tumor cell-remodelled stromal niche remains which provides a supportive microenvironment for clinical relapse^14^. One potential and potent way of a disseminated tumor cell (DTC) to remodel the metastatic niche would be to influence MSC plasticity. Differentiation of MSCs in vitro is mainly induced by chemical cocktails and many signaling pathways and structural components have been reported to contribute to differentiation processes, e.g. the extracellular matrix (ECM), WNT, Notch and microRNAs^15^. Much less is known about factors governing MSC fate in vivo or about the influence of DTCs on MSC stemness, lineage commitment, differentiation and immunomodulatory capacity.

MSCs represent a particular source of ECM molecules such as hyaluronan (HA) that orchestrate regulatory tasks for maintaining the properties of the metastatic niche. In previous studies, it has been shown, that MSCs surround themselves with a coat of HA bound to its receptor CD44 which has been speculated to regulate MSC plasticity. This seems plausible given that CD44 has been reported to be a stem cell marker and is abundant on MSCs, suggesting a functional role in cell–matrix communication^16^.

Here we hypothesize that the ECM component HA modulates MSC plasticity which in turn might alter the cellular composition and cellular interaction of the metastatic niche. We provide evidence that loss of the HA system leads to increased adipogenic differentiation of MSCs which can be reversed by addition of external HA. In addition, we show that metastatic breast cancer cell lines actively seek close proximity to MSCs in dependence of their HA-receptor layilin, positively modulate the MSC HA-system and prevent MSC’s lineage commitment towards adipogenic differentiation. In contrast, glioma cell lines do not alter the MSC-HA matrix. As immunosuppressive properties of MSCs get substantially impaired after adipogenic differentiation, this might provide first hints towards the importance of the hyaluronan MSC matrix in modulating the metastatic niche of different cancer entities.

## Results

A deeper understanding of the interaction between tumor cells and the tumor microenvironment in the metastatic niche is needed to identify key mechanisms explaining the differences in the adaptation to the ‘foreign soil’. Since mesenchymal stem cells are an important factor in orchestrating the bone marrow niche and are at the same time a significant source of extracellular matrix molecules, we investigated the interplay between tumor cells of cancer entities which invariably display tumor cell dissemination but different metastatic behaviors: MDA-MB-321 and U87-MG as model organisms for metastasizing breast cancer and non-metastasizing glioblastoma multiforme, respectively.

### The HA matrix is a negative regulator of adipogenic differentiation of mesenchymal stem cells

**Figure 1:**
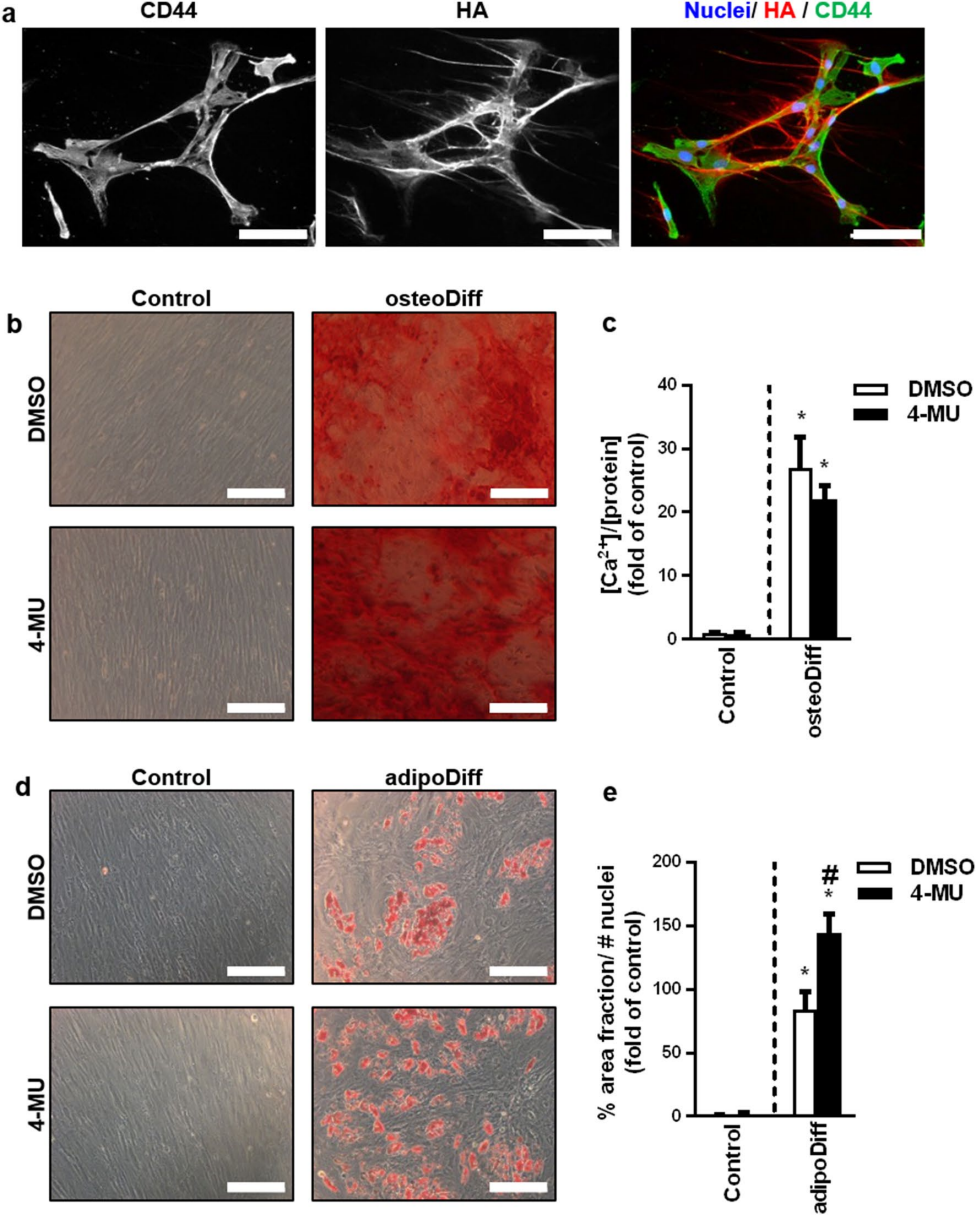
The HA system is strongly expressed in bone marrow-derived mesenchymal stem cells and influences their differentiation potential. (**a**) Cytochemical staining of HA (red) and the stem cell marker and HA interacting receptor CD44 (green). Nuclei were stained with Hoechst 33,342 (blue). Scale bar = 100 µm. (**b**) and (**c**) BmMSCs were differentiated into osteoblasts with or without 4-MU (100 µM) over a period of 28 days. For osteogenic differentiation the cells were stimulated with dexamethasone (10 nM), l-ascorbic acid (50 µM) and β-glycerolphosphate (10 mM) and control treated cells were treated with DMSO for the same period. (**b**) Ca^2+^-phosphate depositions on the cell surface were stained with Alizarin S. Scale bar = 200 µm. (**c**) to quantify osteogenic differentiation, the Ca^2+^—concentration was measured and normalised to the protein concentration of the cell lysate. *p < 0.05 compared to control/DMSO. (**d**) and (**e**) BmMSCs were differentiated into adipocytes with or without 4-MU over a period of 28 days. For adipogenic differentiation the cells were stimulated with dexamethasone (1 µM), insulin (100 µg/ml) and indometacin (200 µM) and control treated cells were treated with DMSO for the same period. (**d**) Lipid vesicles of the adipocytes were stained with Oil Red O. Scale bar = 200 µm. (**e**) Adipogenic differentiation was quantified by determining the area fraction of Oil Red O-stained lipid vesicles and normalised to the number of nuclei. *p < 0.05 compared to control/DMSO; ^#^*p* < 0.05 compared to adipoDiff/DMSO **osteoDiff = **osteogenic differentiation, **adipoDiff = **adipogenic differentiation. n = 4. Mean ± SEM. This figure was prepared with GraphPad Prism (v9.2.0.332, www.graphpad.com).

As a prerequisite for the subsequent experiments, we first determined the presence of HA and its receptor CD44 on the bone marrow-derived MSCs (bmMSCs) utilised in our experiments. As expected, cytochemical stainings revealed a pronounced MSC-derived pericellular HA deposition and a high expression of the HA receptor and stem cell marker CD44 (Fig. 1a).

Within the bone marrow, MSCs mainly differentiate into adipocytes and osteoblasts^17^. To investigate the influence of HA on bmMSC differentiation, bmMSCs were differentiated either into adipocytes or osteoblasts over a period of 28 days with or without simultaneous treatment with 4-methylumbelliferone (4-MU), an inhibitor of HA synthesis. Osteogenic differentiation of MSCs (osteoMSCs) was visualised by staining of Ca^2+^-phosphate depositions with Alizarin Red S (Fig. 1b) and quantified by measuring the Ca^2+^-concentration in the cell layer (Fig. 1c). Inhibition of hyaluronan synthesis did neither lead to spontaneous osteogenic differentiation nor did it affect osteogenic differentiation. For the detection of adipogenic differentiation, lipid vesicles of the adipogenic differentiated bmMSCs (adipoMSCs) were stained with Oil Red O (Fig. 1d), and the area fraction was quantified (Fig. 1e). Inhibition of HA synthesis by 4-MU did not cause spontaneous adipogenic differentiation, even if in some cultures small formations of lipid vesicles could be observed. However, it significantly increased the adipogenic differentiation potential of bmMSCs after chemical stimulation, suggesting a stemness-preserving effect of HA.

**Figure 2:**
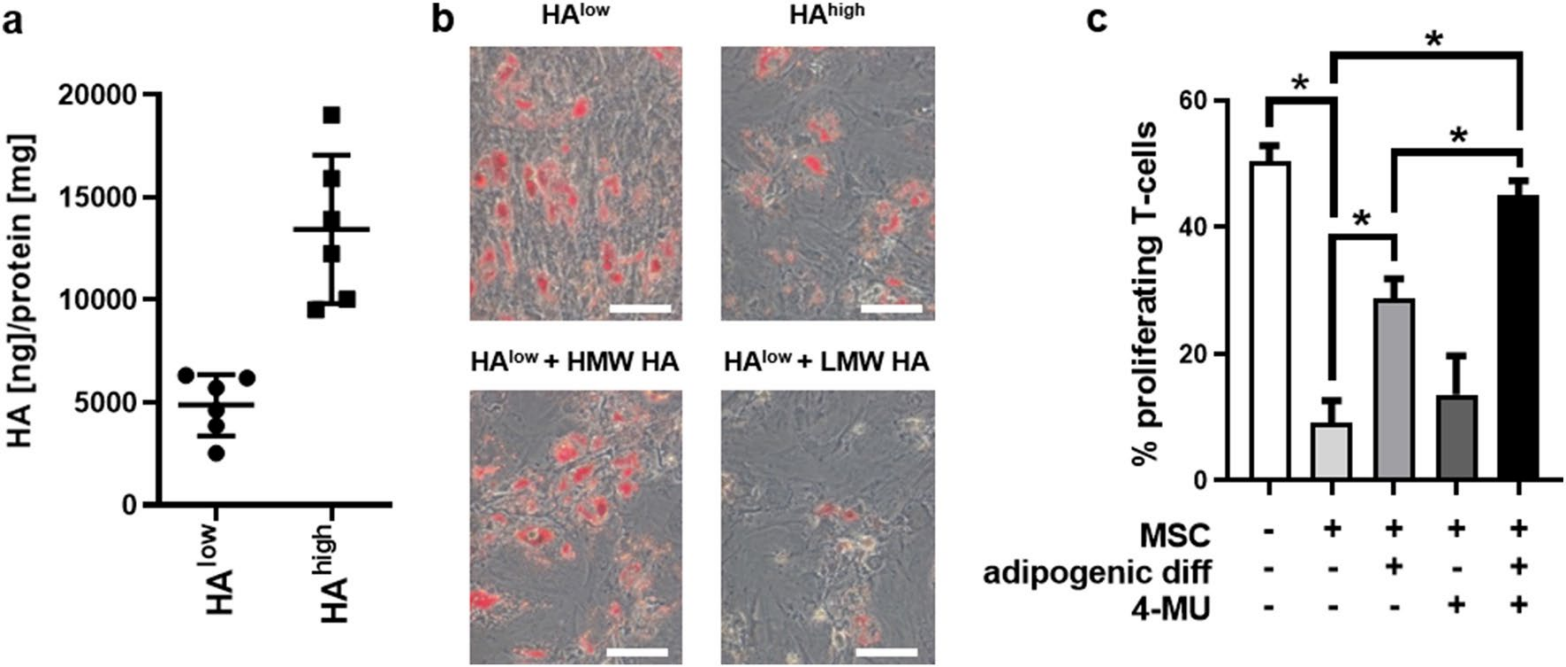
HA impacts on adipogenic differentiation potential of bmMSCs and by this affects their immunosuppressive potential. (**a**) HA-content was measured in the supernatant of different MSC preparations, and two MSC groups (HA^high^ and HA^low^) were detected. (**b**) Representative pictures of bmMSCs belonging to the HA^high^ and HA^low^ group, respectively which were differentiated into adipocytes. BmMSCs from the HA^low^ group were differentiated in the presence of added low molecular weight (LMW-HA) and high molecular weight HA (HMW-HA). Scale bar = 200 µm. n = 4. (**c**) BmMSCs were treated under the experimental condition indicated below the graph over a period of 10 days. Afterwards, the medium was removed, and αCD3 and αCD28 stimulated CD3^+^ T-cells labelled with CFSE were seeded on top of the bmMSC layer. The proliferative rate of T cells was investigated via flow cytometry after 6 days. n = 4. Mean ± SEM. *p < 0.05. This figure was prepared with GraphPad Prism (v9.2.0.332, www.graphpad.com).

Of note, measurement of HA divided individual MSC preparations into two different subgroups (Fig. 2a). We proceeded to investigate whether this difference alters the adipogenic differentiation potential. Indeed, bmMSC with a low amount of HA differentiated more easily into adipocytes (Fig. 2b). Next, we investigated whether extrinsically added HA would reverse this phenotype. Whereas high molecular weight HA (HMW HA) did not change the increased adipogenic differentiation in MSCs with low HA synthetic capacity, low molecular weight HA (LMW-HA) preserved the native MSC phenotype and inhibited adipogenic differentiation (Fig. 2b).

Besides the capability to differentiate into various cell types of the mesodermal lineage, bmMSC exhibit immunomodulatory properties^9^. By using a T-cell proliferation assay, we investigated whether this potential is altered upon adipogenic differentiation. In this experimental setup, αCD3/αCD28 stimulated, CFSE labelled T-cells were seeded on a layer of bmMSC that has been treated for 10 days under various conditions of adipogenic stimulation and/or HA synthesis inhibition. To investigate the maximum proliferative potential of the T-cells, stimulated T-cells were seeded in empty wells (Fig. 2c, 1st bar). The activation of the T-cells was validated by flow cytometry, which showed typical forward scatter/side scatter changes upon activation compared to unstimulated T-cells (Fig. S2). The maximum immunosuppressive potential of the bmMSC was determined by seeding the T-cells on a layer of untreated bmMSCs (Fig. 2c, 2nd bar). T-cells seeded on a layer of pre-adipocytic bmMSCs showed an increased proliferation (Fig. 2c, 3rd bar). This effect was even more pronounced under a simultaneous treatment with 4-MU, which is in line with the observation that 4-MU increases adipogenic differentiation (Fig. 2c, 5th bar). Treatment of bmMSCs with 4-MU lead to a moderate but not significant increase in T-cell proliferation (Fig. 2c, 4th bar). Thus, adipogenic differentiation of MSCs inhibits their T cell suppressive function and this is further enhanced via inhibition of HA synthesis.

### Breast cancer cell-derived factors increase HA matrix and impair adipogenic differentiation of mesenchymal stem cells

As a reduced HA matrix supports adipogenic differentiation, we investigated in a next step, whether cancer cell lines influence the HA matrix of bmMSCs. We chose to compare the effects of the invasive breast cancer cell line MDA-MB-231 and the glioblastoma multiforme cell line U87-MG since both originating tumor entities are known to release disseminated cancer cells into the bone marrow, whereas only breast cancer cells are able to grow efficiently as micrometastases^18, 19^. Thus, we used the two cell lines as models to reveal differences in their interaction with bmMSCs in order to explain their variance in the metastatic potential.

**Figure 3:**
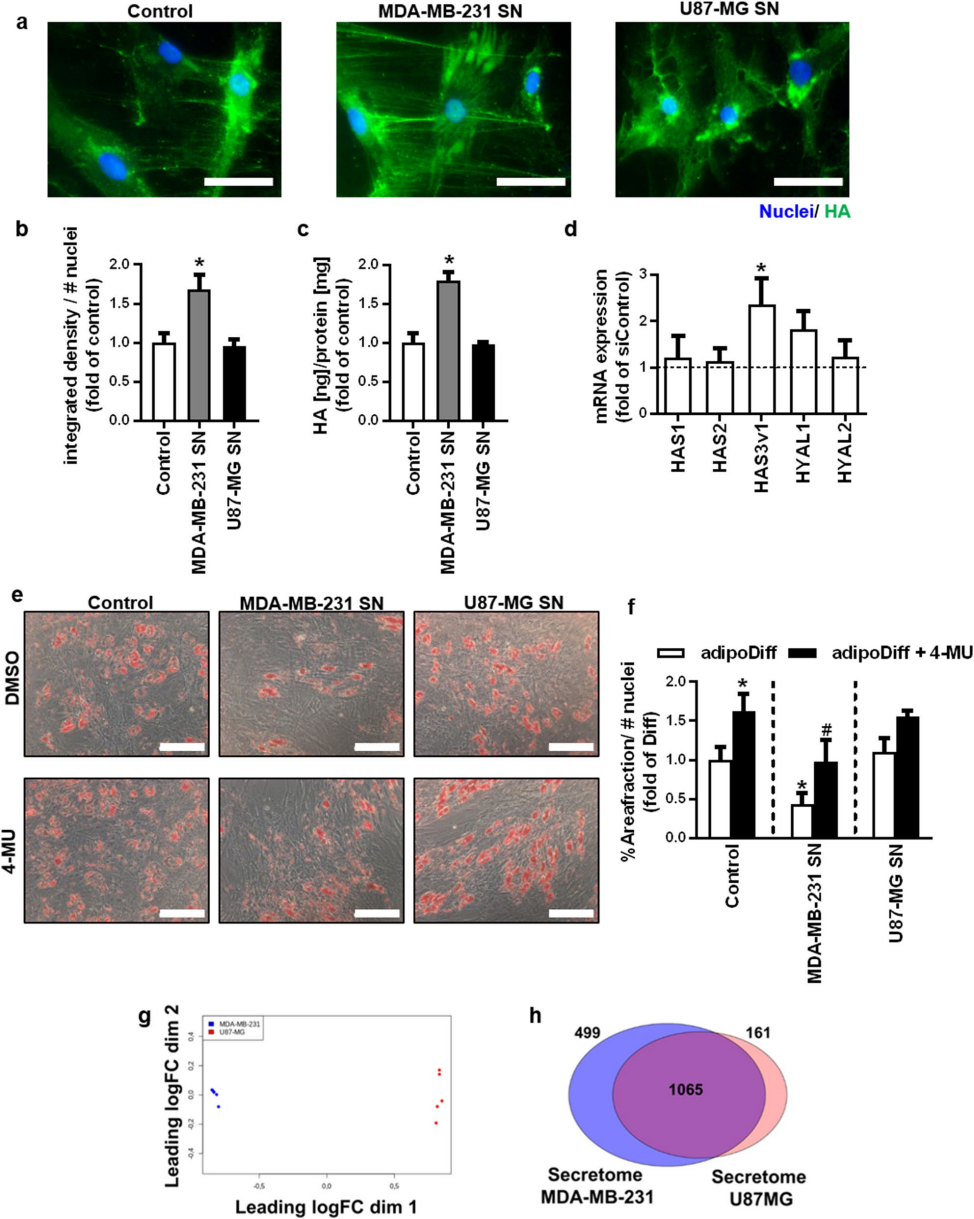
Breast cancer-derived soluble factors increase HA synthesis of mesenchymal stem cells and impair adipogenic differentiation. BmMSCs were incubated with supernatant derived from the cell lines MDA-MB-231 and U87-MG over a period of 72 h. Untreated culture medium was used as control. (**a**) Representative microscope pictures of affinity cytochemical stainings of bmMSCs; HA (green) and Hoechst 33,342 (blue). Scale bar = 100 µm. (**b**) Microscopic pictures were analysed for HA content, measured as integrated density normalised to the number of nuclei. (**c**) The amount of secreted HA was measured with an ELISA-like HABP-binding assay, and the results were normalised to the protein content of the cell lysates. (**d**) BmMSCs were treated with MDA-MB-231 cell-derived supernatant and incubated over a period of 48 h. The expression of HA-system related genes was analysed via qRT-PCR. n = 3. Mean ± SEM. *p < 0.05 compared to control treated bmMSCs. (**e**) and (**f**) BmMSCs were differentiated into adipocytes with or without the addition of 4-MU (100 µM) over a period of 28 days in the presence of cancer cell line-derived supernatant or untreated medium as control. (**e**) Lipid vesicles of adipogenic differentiated bmMSCs were stained with Oil Red O. Scale bar = 200 µm. (**f**) Adipogenic differentiation was quantified by determining the area fraction of Oil Red O-stained lipid vesicles and normalised to the number of nuclei. *p < 0.05 compared to control/adipoDiff; ^#^*p* < 0.05 compared to MDA-MB-231 SN/adipoDiff. n = 4. Mean ± SEM. (**g**) PCA-analysis of supernatants derived by MDA-MB-231 and U87-MG cells analysed by LC–MS, n = 5. (**h**) Venn diagram of secreted and soluble factors in the supernatants of MDA-MB-231 and U87-MG cells investigated via LC–MS. This figure was prepared with GraphPad Prism (v9.2.0.332, www.graphpad.com).

BmMSCs were treated with cancer cell line-derived supernatant (SN) for 72 h, and the HA matrix was investigated via affinity cytochemical stainings. Compared to control-treated bmMSCs, bmMSCs stimulated with MDA-MB-231-derived SN showed a significantly increased HA matrix with a large amount of HA depositions (Fig. 3a, b). This result was confirmed by an ELISA-like HA assay, which revealed a significant increase of secreted HA into the SN after stimulation with MDA-MB-231-derived SN, whereas the amount of secreted HA after treatment with U87-MG-derived SN remained unaltered (Fig. 3c). Hyaluronan is synthesised by three different, transmembranous HA synthase isoforms (HAS 1-3) which extrude the growing HA-strand into the extracellular space^20^. HA is subsequently catabolized mainly by two hyaluronidases (HYAL1 and 2)^21^. We analyzed how the mRNA expression of HAS and HYAL isoforms in bmMSCs are affected by MDA-MB-231 supernatant and found that HAS3, which has been described to synthesize low molecular weight HA, was significantly induced (Fig. 3d).

In a next step, the impact of this differential HA inducing effect of mammary carcinoma vs. glioblastoma cells on the differentiation potential of the stromal cell compartment was employed. The induction of HA synthesis in bmMSCs by MDA-MB-231-derived SN did not influence the osteogenic differentiation of bmMSC, as there were no differences in the Alizarin Red S staining (Fig. S3a, upper panel) and no changes in the Ca^2+^-concentration of the cell layer (Fig. S3b). The simultaneous treatment with 4-MU and osteogenic stimuli did not affect the differentiation potential in both MDA-MB-231 and U87-MG-derived SN (Fig. S3a, lower panel and Fig. S3b). Likewise, the U87-MG derived SN did not affect the adipogenic differentiation of bmMSC (Fig. 3e, f, 5th bar). In contrast to this, MDA-MB-231-derived SN—in parallel to inducing HA synthesis in bmMSC—led to a significant reduction of the adipogenic differentiation after 28 days by quantitative measurements (Fig. 3e, f, 3rd bar). This effect was reversed by the simultaneous treatment with 4 MU, which restored the adipogenic differentiation potential back to control level (Fig. 3e, f, 4th bar), validating the crucial role of HA synthesis in bmMSCs mammary carcinoma supernatant-mediated suppression of adipogenic differentiation. As already shown in Fig. 1d, 4-MU alone did not lead to a spontaneous adipogenic differentiation without further stimuli. These observations were confirmed in direct co-cultures of MDA-MB-231 breast cancer cells with bmMSCs (Fig. S4).

To analyze, which factors, secreted by MDA-MB-231 cells, may induce HA-synthesis of bmMSCs, LC–MS analysis of MDA-MB-231 and U87-MG derived supernatants were performed. As expected, PCA analysis revealed that the secretomes of the investigated cell lines were highly different (Fig. 3g). Expressed in numbers, 499 proteins were significantly higher secreted in MDA-MB-231 derived SN (Fig. 3h). Of note, TGFβ, which has been reported to inhibit adipogenic differentiation^22^ and to be an MSC homing molecule^23^, was among those proteins detected significantly higher in the SN of MDA-MB-231 cells.

**Figure 4:**
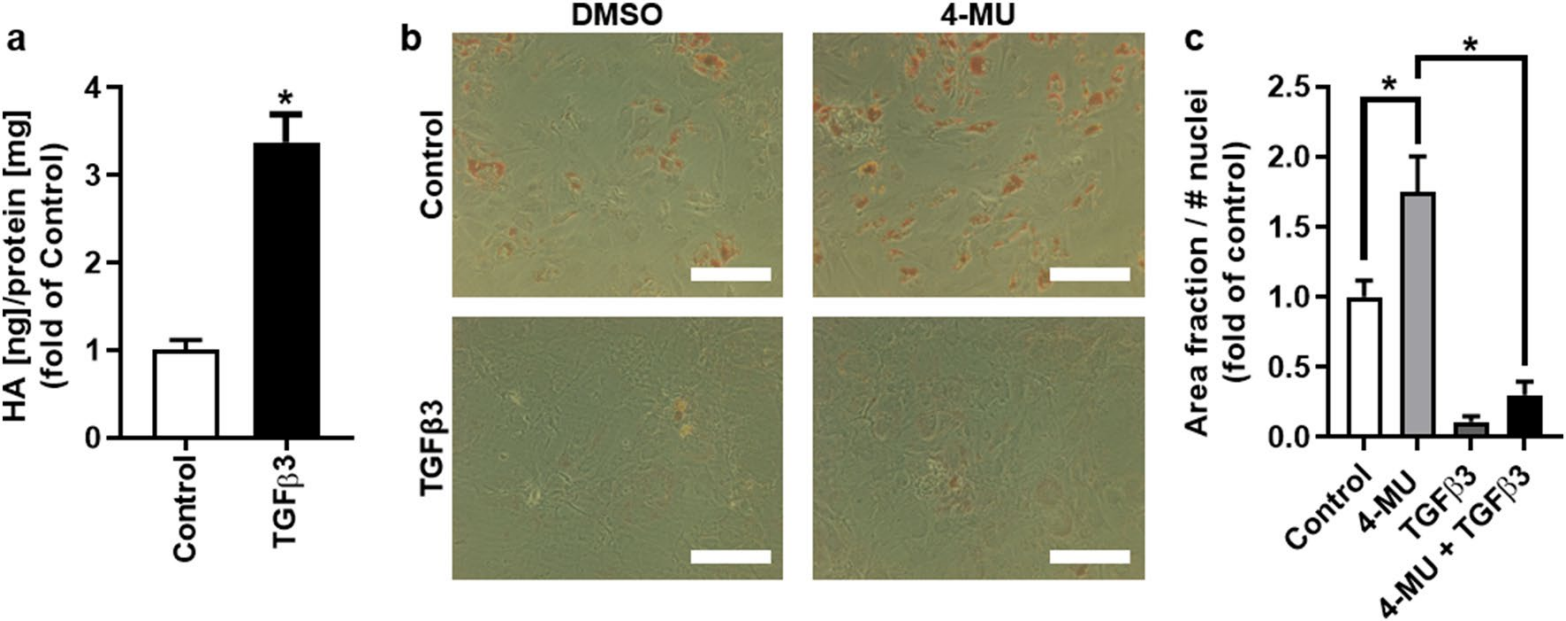
TGFβ3 increases HA-synthesis in bmMSCs and modulates adipogenic differentiation (**a**) bmMSCs were treated with TGFβ3 (5 ng/ml) and the amount of secreted HA was analysed with an ELISA-like HABP-binding assay after 48 h. The results were normalised to the protein content of the cell lysates. n = 4. Mean ± SEM. *p < 0.5 compared to control. (**b**) and (**c**) BmMSCs were differentiated into adipocytes with or without the addition of 4-MU (300 µM) or TGFβ3 (5 ng/ml) over a period of 28 days. (**b**) Lipid vesicles of adipogenic differentiated bmMSCs were stained with Oil Red O. Scale bar = 200 µm. (**c**) Adipogenic differentiation was quantified by determining the area fraction of Oil Red O-stained lipid vesicles and normalised to the number of nuclei. *p < 0.05. n = 4. Mean ± SEM. This figure was prepared with GraphPad Prism (v9.2.0.332, www.graphpad.com).

Indeed, treatment of bmMSCs with TGFβ3 induces HA synthesis in our experimental setup (Fig. 4a). Additionally, the adipogenic differentiation potential was examined in the presence of TGFβ3 and 4-MU simultaneously. Our experiments confirmed the inhibitory effect of TGFβ3 on adipogenic differentiation (Fig. 4b, c) and thereby provide a plausible link between the cancer cell secretome, MSC HA-matrix synthesis and MSC differentiation potential. However, the simultaneous application of 4-MU and TGFβ3 did not restore the adipogenic differentiation potential indicating that TGFβ3 may act downstream of HA induced signaling cascades.

### Breast cancer cells show close interaction with mesenchymal stem cells in dependence of HA

**Figure 5:**
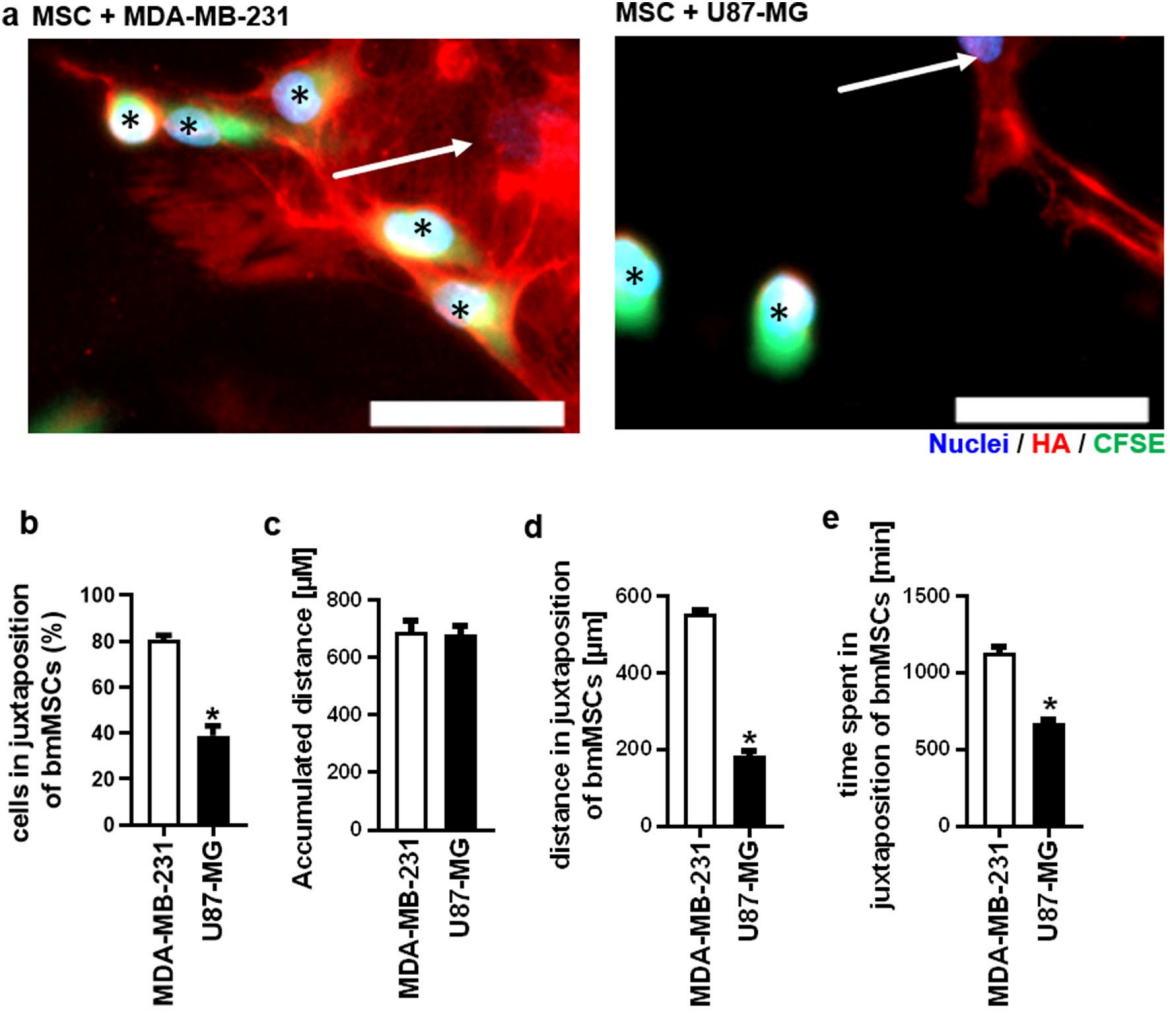
Invasive breast cancer cells interact closely with mesenchymal stem cells. (**a**) Microscopic pictures of a direct co-culture of bmMSCs (arrows) and the cancer cell lines (asterisk) MDA-MB-231 and U87-MG. The cancer cell lines were labelled with CFSE (green). HA was stained with HABP (red) and the nuclei with Hoechst 33,342 (blue). Scale bar = 50 µm. (**b**) Percentage of MDA-MB-231 and U87-MG cells co-localising with bmMSCs. (**c**) The accumulated distance of the tumor cell lines MDA-MB-231 and U87-MG in monoculture was analysed via time-lapse microscopy over a period of 24 h. (**d**) and (**e**) Time-lapse analysis of MDA-MB-231 and U87-MG cells cultivated in co-culture with bmMSCs. (**d**) Accumulated distance and (**e**) the time spent in juxtaposition of bmMSCs**.** n = 4. Mean ± SEM. *p < 0.05 compared to MDA-MB-231. This figure was prepared with GraphPad Prism (v9.2.0.332, www.graphpad.com).

It was recently reported that MSCs might play a crucial role in the invasion of invasive breast cancer cells into the bone marrow^24^. Thus, we investigated the mutual interaction between bmMSCs and cancer cell lines in direct co-cultures. Cancer cells were stained with CFSE prior to seeding to allow differentiation between cancer cells and bmMSC. Co-cultures were incubated over a period of 48 h followed by staining for HA. While MDA-MB-231 breast cancer cells were located in close proximity to the bmMSC U87-MG glioblastoma cells were much more randomly distributed, thus exhibiting a significantly lower co-localisation with bmMSC (Fig. 5a, b). In addition, bmMSC also revealed a decreased HA matrix when co-cultivated with U87-MG cells compared to co-cultivation with MDA-BM-231 cells (Fig. 5a).

Time-lapse microscopy and manual tracking of the cells revealed no differences in the accumulated distance between MDA-MB-231 and U87-MG cells over a period of 24 h when grown in monoculture (Fig. 5c). Next, both cancer cell lines were independently co-cultivated with bmMSCs for 24 h. To quantify the co-localisation between tumor cells and bmMSC, only tumor cells in juxtaposition to bmMSC were tracked and the time and migrated distance were recorded (Fig. 5d, e). Interestingly, the time and distance of MDA-MB-231 cells spent in direct juxtaposition of bmMSCs was significantly higher than of U87-MG cells, indicating that MDA-MB-231 breast cancer cells actively seek and migrate towards bmMSC cells in vitro (Fig. 5d, e).

### Adipogenic differentiation of mesenchymal stem cells inhibits their adhesive potential

Hypothesising that close proximity to MSCs might be the preferred and potentially beneficial location of cancer cells within the BM microenvironment we next investigated whether cancer cells show a preference for interaction with native or adipogenic differentiated MSCs (adipoMSCs).

**Figure 6:**
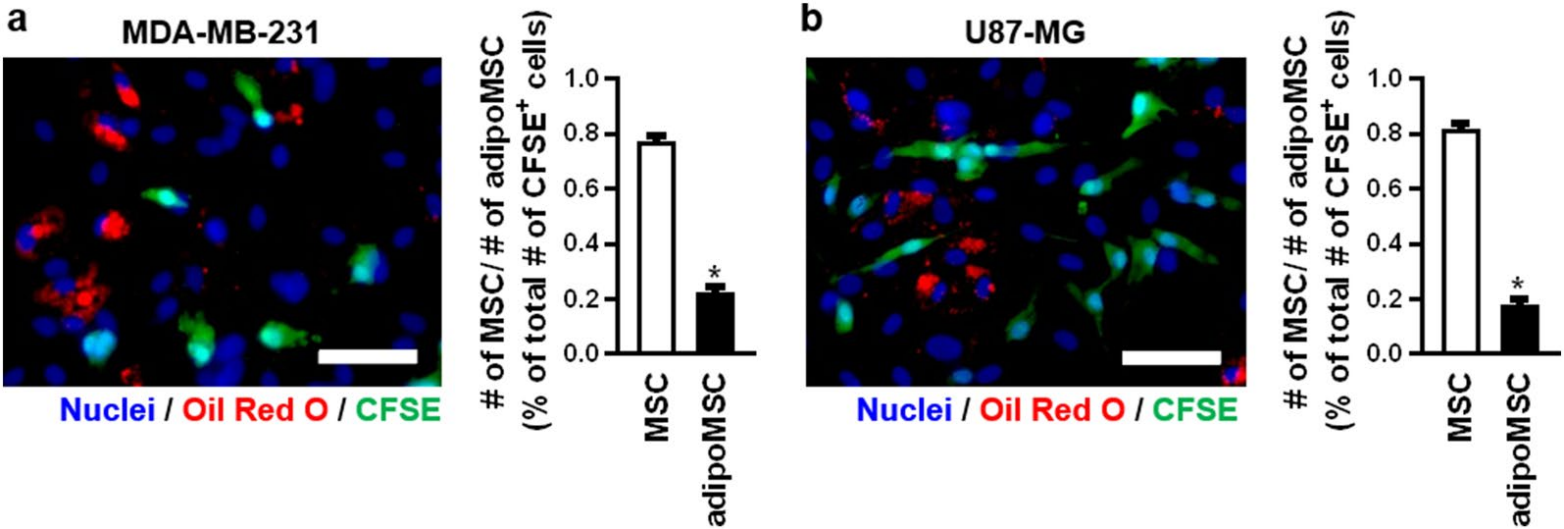
Adipogenic differentiated mesenchymal stem cells (adipoMSC) show less interaction with cancer cells. The CFSE-labelled cancer cell lines MDA-MB-231 and U87-MG were seeded on a confluent layer of adipogenic differentiated bmMSCs with a percentage of differentiation of approximately 50% and incubated for 24 h. Number of differentiated bmMSCs was microscopically evaluated. Nuclei were stained with Hoechst 33342 (blue), and lipid vesicles of adipogenic differentiated bmMSCs were stained with Oil Red O (red). The localisation of the cancer cells (blue and green) in comparison to native bmMSCs (blue; MSCs) or adipogenic differentiated bmMSCs (blue and red; adipoMSC) was investigated. Cancer cells were considered to be co-localised with bmMSCs when the CFSE signal was located in the area of a nucleus of a bmMSC + 5 µm. (**a**) and (**b**) Representative microscopic images and its quantification of the co-localisation of the cancer cell lines (**a**) MDA-MB-231 and (**b**) U87-MG either with native bmMSCs or adipogenic differentiated bmMSCs. Scale bar = 100 µm. For each n five microscopic pictures were analysed. n = 4. *p < 0.05 compared to MSC. This figure was prepared with GraphPad Prism (v9.2.0.332, www.graphpad.com).

For this purpose, CFSE labelled cancer cells were seeded on a confluent layer of bmMSCs with an aprroximately 50% grade of adipogenic differentiation. After 24 h the cells were fixed and stained with Oil Red O. Microscopic investigations were used to analyse whether the tumor cells show a preference in terms of their interaction partners and either co-localised with native bmMSCs (Oil Red O negative) or adipoMSCs (Oil Red O positive). Both MDA-MB-231 and U87-MG cells interacted to a greater extent with native bmMSC compared to adipoMSCs (Fig. 6a, b).

### The hyaluronan system mediates the interaction between breast cancer cells and mesenchymal stem cells via the HA receptor layilin

Given the increased HA synthesis of MSC in response to MDA-MB-231 breast cancer SN and that HA is able to connect cells via binding of the HA strands to its receptors (CD44, RHAMM, LAYN) we went on to investigate whether a transmembranous HA receptor on the MDA-MB-231 cells might be responsible for the communal migration of the two cell types.

**Figure 7:**
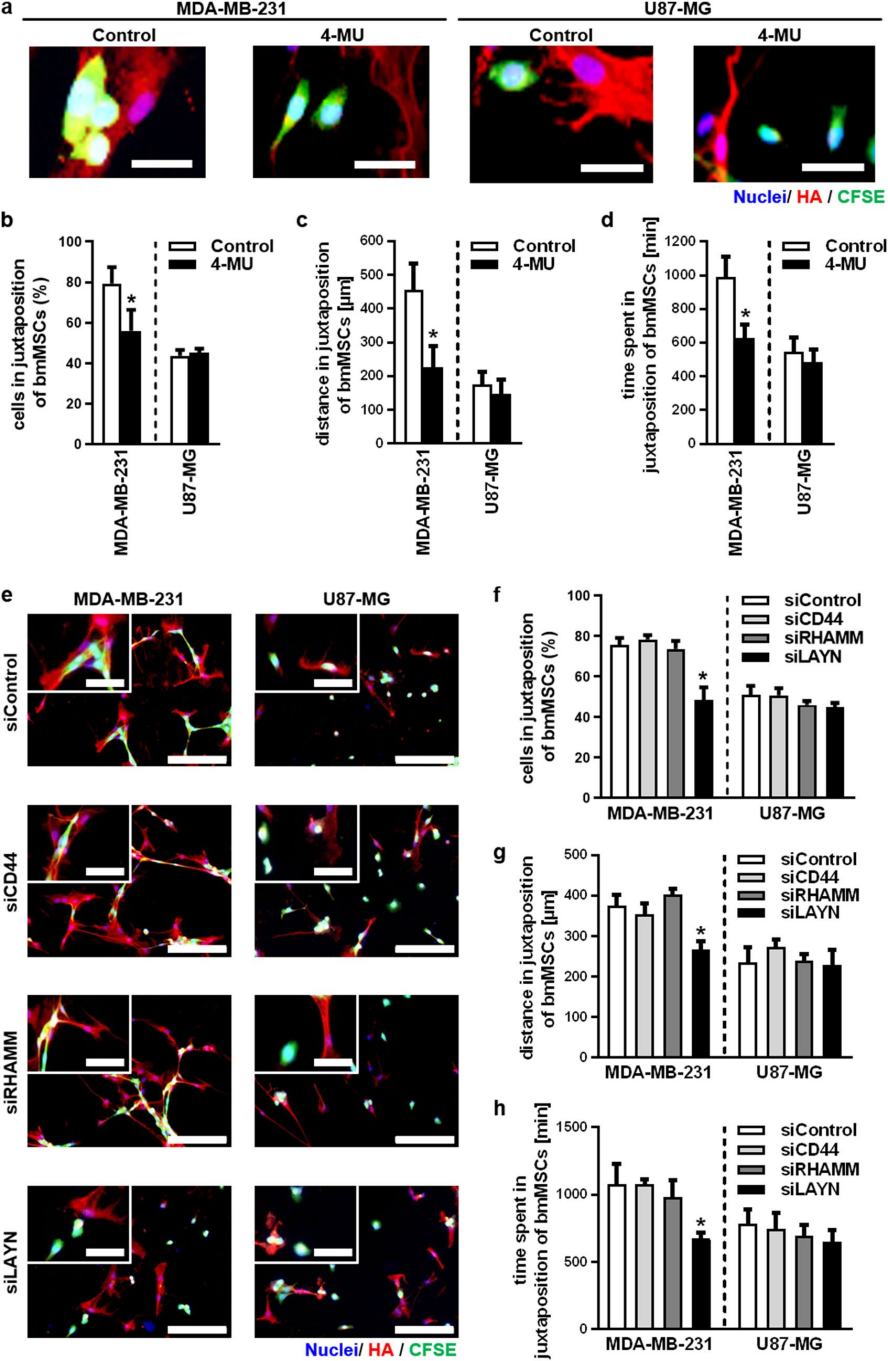
Intimate interaction between invasive breast cancer cells and mesenchymal stem cells is mediated by the hyaluronan system via layilin. To investigate the role of the hyaluronan system in the interaction between cancer cells and bmMSCs either the HA synthesis of the bmMSCs was inhibited with 4-MU (100 µM) or by silencing the expression of the HA interacting receptors in the cancer cell lines MDA-MB-231 and U87-MG via siRNA. (**a**) Representative cytochemical stainings of a direct co-culture of bmMSCs and CFSE-labelled MDA-MB-231 and U87-MG cells (green). BmMSCs were previously treated with 4-MU (100 µM) over a period of 72 h. Cells were stained 48 h after the co-cultures were established. Scale bar = 50 µm. (**b**) Percentage of MDA-MB-231 and U87-MG cells co-localising with bmMSCs after bmMSCs were treated with 4-MU (100 µM). (**c**) The accumulated distance and (**d**) the time spent in juxtaposition of bmMSCs as quantified by time-lapse microscopy. (**e**) Representative cytochemical stainings of a direct co-culture of bmMSCs and HA receptor-depleted cancer cells. The expression of the HA interacting receptors in MDA-MB-231 and U87-MG cells was silenced via siRNAs directed against CD44, RHAMM and LAYN. Afterwards, the cancer cells were stained with CFSE (green). Scale bar = 200 µm, inset = 50 µm. (**f**) Analysis of the percentage of MDA-MB-231 and U87-MG cells co-localising with bmMSCs. Time-lapse quantification of (**g**) accumulated distance and (**h**) time spent in juxtaposition of HA receptor depleted cancer cells. n = 4. Mean ± SEM. *p < 0.05 compared to the respective control treated cells. This figure was prepared with GraphPad Prism (v9.2.0.332, www.graphpad.com).

The role of the HA system in the interaction between cancer cells and bmMSCs was evaluated in direct co-cultures. For this reason, HA synthesis of bmMSCs was inhibited by 4-MU. The treatment of bmMSCs with 4-MU reduced the HA signal in affinity cytochemical stainings and removed the reticular structures observed in co-culture with MDA-MB-231 cells (Fig. 7a). As a consequence, the interaction of MDA-MB-231 cells with bmMSCs was significantly decreased in comparison to control treated bmMSCs (Fig. 7a, b). Furthermore, analysis of microscopic time-lapse recordings revealed a reduction of the distance travelled and time spent in juxtaposition of bmMSCs (Fig. 7c, d; Fig. S5a). Contrary to the findings in the MDA-MB-231 cell line, both the co-localisation and the motility in juxtaposition of bmMSCs after 4-MU treatment of U87-MG cells remained unaffected (Fig. 7a–d, Fig. S5a).

To identify the HA receptor that mediates binding of tumor cells to bmMSCs in our experimental setup, we next silenced the HA receptor genes CD44, RHAMM and LAYN by siRNA. qRT-PCR analysis was used to validate the decrease of gene expression (Fig. S6a–c). The knockdown of these receptors neither influenced the cell number (Fig. S7d) nor the motility of MDA-MB-231 and U87-MG cells in monoculture (Fig. S7e, f). In the case of the cell line MDA-MB-231, siLAYN specifically reduced co-localisation with bmMSCs (Fig. 7e, f), accumulated distance (Fig. 7g, Fig. S5b), and time in juxtaposition in co-localisation with bmMSCs (Fig. 7h). In contrast to this observation, both the co-localisation and motility in juxtaposition of bmMSCs were not changed when the expression of CD44, RHAMM and LAYN was repressed in U87-MG cells (Fig. 7e–h).

Taken together, these data indicate that the adhesion of the breast cancer cell line MDA-MB-231 to bmMSC is mediated by the HA receptor layilin and in turn results in reduced motility of cancer cells in the presence of undifferentiated bmMSCs.

## Discussion

Our experiments provide novel insights into tumor cell–stroma interactions that may explain the differences in metastatic potential of different tumor entities. We show that metastatic breast cancer cells but not glioblastoma cells induce the extracellular matrix component HA in human bmMSCs. This process may be utilized by certain tumor entities to maintain a supportive metastatic niche since HA inhibits adipogenic differentiation of human bmMSCs thus maintaining their immunosuppressive and pro-tumorigenic properties. Additionally, this mechanism fosters close cellular interaction between tumor cells and bmMSCs, which depends on the binding of the HA matrix to the HA receptor LAYN.

To the best of our knowledge, our study is among the first to analyze the impact of the HA system on differentiation potential of primary human bone marrow-derived MSCs. In subcutaneous tissue, HA exerts opposing effects on adipogenesis via its two main receptors CD44 and RHAMM^25^. In detail, CD44 is a prerequisite for subcutaneous adipogenesis, while RHAMM prevents this process^26^. Our results imply that induced HA synthesis impairs adipogenesis potentially via RHAMM activation in bmMSCs. This hypothesis was further corroborated by the finding that supernatant derived from MDA-MB-231 cells increase HA synthesis and subsequently inhibited the adipogenic differentiation of MSCs, while U87-MG supernatant did not influence either. This impact of HA on adipogenic differentiation was confirmed by the fact that the impaired adipogenesis was reverted by treatment with the HA synthesis inhibitor 4-MU. These findings are well in line with recent studies which showed a connection between HA and the maintenance of the stemness of placenta-derived stromal cells^27^.

The size of HA has previously been reported to influence its signaling properties^28^. Presumably, HA needs a minimal molecular size to engage to its receptors, further size increase should, in theory, have little effect on receptor recognition. However, convincing evidence for size-dependent effects has not been provided in the past. A possible explanation for this fact might be that a difference in HA size mediates the complex and variable clustering of HA-receptors with other binding partners, e.g. cMET, PDGFR and integrins^29, 30^. Interestingly, in our experimental setting only low molecular weight HA was able to maintain MSC stemness whereas high molecular weight HA had no effect. MDA-MB-231 cells increased the expression of HAS3 which has been described to synthesize smaller sized HA compared to the other HAS isoforms. In addition, upregulation of HYAL1 was detected which is capable of facilitating the production of low molecular weight HA from larger precursors. Based on our findings we hypothesize that directed modulation of the MSC HA-matrix by the tumor cells might lead to an increased stemness of MSCs in the metastatic niche.

An important step in the population of the pre-metastatic niche by tumor cells is the adhesion to the target site. The HA matrix derived from bmMSCs represents a possible conducive soil. The importance of HA and its interaction with the HA interacting receptor CD44 in the process of extravasation is known to recruit activated T-lymphocytes to the site of inflammation^31^. Our experiments translate these findings to the stromal cells compartment and indicate that HA-inducing MDA-MB-231 cells interact significantly stronger with bmMSC, which was abolished by the treatment of bmMSCs with 4-MU. By performing knockdown experiments, we identified that this interaction is mediated by LAYN. So far, little is known about the function of LAYN, however, a role of LAYN in cell adhesion and motility has been proposed^32^. Correlating with our data, inhibition of LAYN expression resulted in a significant reduction of lymphatic metastasis of A549 lung cancer cells in vivo^33^*.* Furthermore, LAYN was recently considered as a prognostic marker and a high LAYN expression correlated with increased lymphatic metastasis and a decreased survival of patients with gastric and colon cancer^34^. Interestingly, the interaction between U87-MG cells and bmMSCs remained unaltered after knockdown of LAYN. This observation may be explained by the generally low interaction between these two cell types and the fact that the HA synthesis of bmMSCs is not induced by U87-MG cells. A possible therapeutic approach derived from the results from our study could be based on the close interaction between bmMSCs and invasive breast cancer cells and on the tumor-trophic migratory properties of MSCs shown in previously published studies^28^. Hence, engineered MSCs may be considered as drug delivering vectors who specifically target cancer cells and provide anti-tumoral and/or anti-metastatic effects.

Another critical aspect for tumor cell survival is the escape from immunosurveillance. BmMSCs can modulate the immune system by reducing the proliferation, interferon-γ production and cytotoxicity of CD4^+^ and CD8^+^ cells, eventually leading to the conversion of these cells into regulatory, immunosuppressive T-lymphocytes^9, 35^. Thus, MSCs are able to suppress the immune response and have been shown to alleviate allogeneic graft-versus-host (GvH) reactions and to provide pro-tumorigenic effects in murine in vivo models^36, 37^. The differentiation of MSCs can change their influence on other tissues; osteoblasts derived from MSCs positively influence hematopoiesis^38^, while adipocytes are negative regulators of the hematopoietic microenvironment^39^. Adipogenic differentiation of MSCs was shown to alter the immunosuppressive properties in inflamed adipose tissue in the sense that suppression of neutrophil recruitment was abolished after adipogenic differentiation^40^. In the T-cell inhibition assay, we demonstrated a reduced immunosuppressive potential of the bmMSC on activated T-cells after adipogenic differentiation. An enhanced adipogenic differentiation by HA synthesis inhibition via 4-MU resulted in a further reduction of the immunosuppressive potential. This observation provides a new mechanism how inhibition of adipogenic differentiation supports escape from immunosurveillance. These findings may provide an explanation why the only reported cases of brain cancer patients, which show extracranial metastasis, are patients with profound immunosuppression due to radio- and chemotherapy^41, 42^ as U87-MG are not able to maintain the immunosuppressive function of bmMSC. Our data suggest that a reduced adipogenic differentiation might preserve the pro-tumorigenic properties of bmMSC.

In conclusion, we propose that invasive breast cancer cells interfere with the adipogenic differentiation potential of bmMSCs via the induction of HA in bmMSC mediated in part by TGFβ3. Thus, MDA-MB231 cells create their own pro-metastatic microenvironment by preserving pro-tumorigenic properties of undifferentiated bmMSCs which potently support tumor cell proliferation, mediate intimate tumor cell-bmMSC co-localisation and interaction and provide immuno-suppressive effects. The interaction of tumor-associated MSCs with tumor cells has been recognised as a promising emerging target^43^. Our study indicates that targeting HA binding proteins such as LAYN on metastatic cancer cells or interference with the HA-modulating effect of tumor cells on MSC via antagonizing TGFβ3 might provide novel strategies to prevent the dissemination and outgrowth of metastatic cancer cells in the metastatic niche.

## Materials and methods

### Reagents

If not other indicated all reagents were purchased from Sigma-Aldrich (St. Louis, MO, USA).

### Isolation, culture and characterisation of human bone marrow-derived mesenchymal stem cells (bmMSCs)

Five to ten ml of heparinized human bone marrow aspirate obtained from healthy donors after informed consent were treated with red cell lysis buffer. Afterwards the cells were washed with HBSS (Lonza, Basel, Switzerland) and resuspended in 0.2 ml/cm^2^ DMEM + 1 g/l glucose (Lonza, Basel, Switzerland) supplemented with 2 mM l-glutamine (Lonza, Basel, Switzerland), 5% human fresh frozen plasma (FFP) and 5% platelet lysate (both provided by the Institute of Haemostaseology and Transfusion Medicine, University Hospital, Düsseldorf). Cells were transferred to a 175 cm^2^ tissue culture flask (Corning, Corning, NY, USA) in a density of ≤ 10^6^ cells/cm^2^ and incubated at 37 °C and 10% CO_2_. BmMSCs were subcultivated at a density of 2000–4000 cells/cm^2^ into CellStack^®^-1 chambers. At a confluence of 80%, cells were again subcultivated in CellStack^®^-1 and CellStack^®^-5 chambers until end of passage 2.

BmMSCs displayed a characteristic immunophenotype with > 90% CD73^+^ CD105^+^ cells and < 1% of CD3^+^ CD45^+^ cells (Fig. S1). For characterisation, the following antibodies were used: CD73 (BD Biosciences, Heidelberg, Germany), CD105 (Ancell, Hamburg, Germany), CD45 (Beckmann Coulter, Krefeld, Germany), and CD3 (Beckmann Coulter). For experiments, bmMSCs in passage 3–5 were used.

The ethics committee of the Medical Faculty of the Heinrich-Heine-University, Düsseldorf (No. 1830), approved the generation of MSCs from healthy volunteer bone marrow donors for research purposes.

### T-lymphocyte inhibition assay

Human T-lymphocytes were isolated from healthy blood donors by erythrocyte-rosetting gradient centrifugation, using sheep red blood cells. Purified T-lymphocytes (> 90% CD3^+^) were labelled with 0.5 µM 5(6)-Carboxyfluorescein *N*-hydroxysuccinimide ester (CFSE; Life Technologies, Carlsbad, CA, USA) as described in the section CFSE staining. Stained T-cells were stimulated in triplicates with 1 µg/ml anti-CD3 and 1 µg/ml anti-CD28 (both BD Pharmingen, Heidelberg, Germany) and cultivated in 96-well plates. To investigate the immunoregulatory effect of bmMSCs, cells were seeded in a ratio bmMSCs:T-cells of 1:5 and the proliferative rate was measured after 6 d via flow cytometry, using a BD FACSCalibur™ (Becton Dickinson, Franklin Lakes, NJ, USA).

### Cultivation of cancer cell lines

The mammary cancer cell line MDA-MB-231 (RRID: CVCL_0062) was purchased from CLS cell line service GmbH (Eppelheim, Germany) and the glioblastoma cell line U87-MG (RRID: CVCL_0022) was obtained from ATCC (ECACC, Porton Down, UK). The identity of these cell lines was validated by short tandem repeat (STR) analysis, performed by the Institute of Forensic Medicine, University Hospital Duesseldorf. All cell lines were cultivated in DMEM + 1 g/l glucose + 10% fetal calf serum + 100 U/ml penicillin–streptomycin and incubated at 37 °C with 5% CO_2_ in a humid microenvironment.

### Generation of supernatant

Cells were seeded in a density of 5000 cell/cm^2^ in T75 flasks (Greiner Bio-One, Kremsmünster, Austria) in 12 ml cultivation medium and incubated for 72 h at 37 °C and 5% CO_2_. The supernatant was sterile filtered with a Filtropur S filter (Sarstedt, Nümbrecht, Germany) with a pore size of 0.2 µm and used immediately.

### Stimulation of bmMSCs with hyaluronic acid

The cells were treated with 100 µg/ml HEALON^®^ 5 (AMO Germany GmbH, Ettlingen, Germany) high molecular weight (HMW) HA with a molecular weight of 4000 kDa. For the generation of low molecular weight (LMW) HA HMW-HA was diluted 1:1 with PBS and sonicated in a pre-warmed (60 °C) Ultrasonic bath for 3 h. Stock concentration of both HMW-HA and LMW-HA was 5 mg/ml.

### Quantification of secreted hyaluronic acid

The supernatant was transferred into 1.5 ml microtubes and centrifuged for 5 min at 300 rcf. The pellet was discarded, and the supernatant was analyzed with the Hyaluronic Acid Test Kit (Corgenix, Westminster, CO, USA) according to the manufacturer’s manual. Hyaluronic acid was detected at a wavelength of 800 nm. The cell layer was lysed with 0.1 M NaOH and incubated for 15 min at RT. The protein concentration was determined with the BCA Protein Assay Kit (Thermo Fisher Scientific, Waltham, MA, USA). Amount of the hyaluronic acid in the supernatant was normalized to the protein concentration.

### Adipogenic and osteogenic differentiation of mesenchymal stem cells

BmMSCs were seeded in a density of 8000 cells/cm^2^ and cultivated as described above. When the cells reached a confluency of ~ 80% the medium was exchanged with DMEM + 1 g/l glucose + 10% FCS + 100 U/ml PenStrep with 2 mM l-glutamine. For adipogenic differentiation, the medium contained following stimuli: dexamethasone (1 µM), insulin (100 µg/ml) and indometacin (200 µM). For osteogenic differentiation, the medium contained dexamethasone (10 nM), l-ascorbic acid (50 µM) and β-glycerolphosphate (10 mM). Adipogenic bmMSCs were stained with Oil Red O, and osteogenic bmMSCs were stained with Alizarin S.

### Oil Red O staining and quantification of adipogenic differentiation

Cells were fixed with 4% PFA for 15 min and washed with 60% isopropanol (VWR, Radnor, PA, USA). Cells were then stained for 10 min at RT with a 0.2% Oil Red O solution. Afterwards, the cells were washed with PBS, and the nuclei were stained with a 1:1000 dilution of Hoechst 33342 (Invitrogen, Carlsbad, CA, USA). The degree of differentiation was analysed by a microscopic approach using an Axio Observer.Z1 (Carl Zeiss, Oberkochen, Germany). The area fraction of the Oil Red O staining was normalised to the number of nuclei with the freely available software Fiji^44^.

### Alizarin Red S staining of osteogenic differentiation

Cells were fixed with 4% PFA for 15 min and washed with ddH_2_O. Afterwards, the cells were incubated with 2% Alizarin S (pH 4.0) for 10 min and washed gently with PBS. Staining was analyzed via brightfield microscopy.

### Calcium assay

Osteogenic differentiation was quantified by measuring the Ca^2+^-concentration of the cell layer. Therefore, the Calcium Assay Kit (Abnova, Taipeh, China) was used according to the manufacturer’s manual. Results were normalised to the protein content of the samples.

### Sample preparation for secretome analysis

For mass spectrometric analysis 11 ml of conditioned medium were centrifuged at 4 °C, 5 min at 1000×*g*, and the supernatants were sterile-filtered (pore size: 0.2 μm Acrodisc MS syringe filter, Pall, Dreieich, Germany) to remove cell debris and death cells. The proteins in the conditioned medium were precipitated for 1 h at 4 °C by addition of 50% (w/v) trichloroacetic acid and 0.1% sodium [dodecanoyl(methyl)amino]acetate in water, sedimented by centrifugation and washed with ice-cold acetone. After repeated centrifugation, the protein pellet was shortly airdried at room temperature and resolved in 50 μl of lysis buffer (30 mM tris(hydroxymethyl)aminomethane, 2 M thiourea, 7 M urea, and 4% (w/v) 3-[(3-cholamidopropyl)dimethylammonio]-1-propanesulfonate, pH 8.5). Proteins from frozen cell pellets were extracted as described elsewhere^45^. Briefly, cells were lysed and homogenized in lysis buffer with a TissueLyser (Qiagen, Hilden, Germany) and supernatants were collected after centrifugation for 15 min at 14,000×*g* and 4 °C, supernatants were collected. Protein concentration was determined by means of Pierce 660 nm Protein Assay (Fischer Scientific, Schwerte, Germany) and 10 µg protein per sample were loaded on a SDS-PAGE for in-gel-digestion. The isolated gel pieces were reduced (50 µl, 10 mM DTT), alkylated (50 µl, 50 mM iodoacetamide) and underwent afterwards tryptic digestion (6 µl, 200 ng trypsin in 100 mM ammonium bicaonate). The peptides were resolved in 15 µl 0.1% trifluoracetic acid and subjected to liquid chromatography.

### LC–MS analysis

For the LC–MS analysis a QExactive plus (Thermo Scientific, Bremen, Germany) connected with an Ultimate 3000 Rapid Separation liquid chromatography system (Dionex/Thermo Scientific, Idstein, Germany) equipped with an Acclaim PepMap 100 C18 column (75 µm inner diameter, 25 cm length, 2 mm particle size from Thermo Scientific, Bremen, Germany) was applied. The length of the isocratic LC gradient was 120 min. The mass spectrometer was operating in positive mode and coupled with a nano electrospray ionization source. Capillary temperature was set to 250 °C and source voltage to 1.4 kV. In the QExactive plus mass spectrometer for the survey scans a mass range from 200 to 2000 *m/z* at a resolution of 70,000 was used. The automatic gain control was set to 3,000,000 and the maximum fill time was 50 ms. The 10 most intensive peptide ions were isolated and fragmented by high-energy collision dissociation (HCD)^46^.

### Computational mass spectrometric data analysis

Peptide and protein identification and quantification was done by using MaxQuant (version 1.6.2.10, MPI for Biochemistry, Planegg, Germany) applying standard parameters. As human samples were analyzed, searches were conducted using a specific proteome database (human Swissprot, downloaded 02/19/18) from UniProt. Methionine oxidation and acetylation at protein N-termini were set as variable modification and carbamidomethylations at cysteines were considered as fixed modification. Peptides and proteins were accepted with a false discovery rate set to 1%. Unique and razor peptides were used for label-free quantification and peptides with variable modifications were included in the quantification. The minimal ratio count was set to two and the matched between runs option was enabled.

The normalized intensities as provided by MaxQuant were analyzed by using Perseus framework (version 1.5.0.15, MPI for Biochemistry, Planegg, Germany). Only proteins containing at least two unique peptides and a minimum of 4 valid values in each group were taken into consideration for protein quantification. Proteins which were identified only by site or marked as contaminant (from the MaxQuant contaminant list) were excluded from the analysis. For the calculation of enriched proteins in the two groups a Student’s t-tests was applied (*p* ≤ 0.01)^46^.

### Cytochemical HA staining

Cells were grown on coverslips. At the endpoint of the experiment, the medium was removed, the cells were washed with pre-warmed PBS and fixed with an acidic fixation solution (70% EtOH, 4% PFA and 5% acetic acid in ddH2O) for 15 min at RT. The cells were washed three times with PBS for 5 min and blocked with 5% bovine serum albumin (BSA) in PBS at RT on a rocking plate for at least 1 h. The samples were incubated with 0.4 µl HABP (Calbiochem, San Diego, CA, USA) per 100 µl 2.5% BSA in PBS and incubated in a moist chamber overnight. Streptavidin-Cy3 (Invitrogen, Karlsruhe, Germany) was used as a secondary in a dilution of 1:1000 in PBS. The nuclei were stained with Hoechst 33342 (Invitrogen, Karlsruhe, Germany). The coverslips were embedded in ProlongTM Gold (Invitrogen, Karlsruhe, Germany) and sealed with nail polish. Microscopic pictures were made with an Axio Observer.Z1 (Carl Zeiss, Oberkochen, Germany).

### CFSE staining

To assess the proliferative rate, cells were stained with CFSE. Therefore, 1 × 10^6^ cells were resuspended in 1 ml PBS containing 0.1% FCS. 2 µl of a 5 mM CFSE stock solution was directly added to the cell suspension and incubated at 37 °C for 15 min. The reaction was stopped by adding 5 ml of DMEM + 10% FCS + 1% FCS + 100 U/ml, and centrifuged at 300 rcf for 5 min. Afterwards, the cells were washed twice with PBS and seeded in a density of 12,500 cells/cm^2^. The proliferative rate was determined via flow cytometry by measuring the mean signal intensity at a wavelength of 525/30 nm using an EasyCyte 5 (Millipore, Burlington, MA, USA).

### Time-lapse microscopy and manual tracking of cells

Time-lapse microscopy was performed with an Axio Observer.Z1 (Carl Zeiss, Oberkochen, Germany) equipped with an incubation XL multi S1 (Pecon, Erbach, Germany) at 37 °C and 5% CO_2_. Pictures were taken over a period of 24 h and an interval of 15 min. Recordings were analysed using the plug-in “manual tracking” of Fiji^44^. The acquired data were further analysed with the “Chemotaxis and Migration Tool” by ibidi (Planegg, Germany).

### SiRNA transfection

To reduce the gene expression, tumor cells were reverse transfected with siRNAs directed against specific genes. Therefore, 10 nM siRNA was incubated with 6 µl Lipofectamine^®^ RNAiMAX (Thermo Fisher Scientific, Waltham, MA, USA) in 200 µl of serumfree medium for 20 min. Afterwards, 5 × 10^4^ cells were added. After 24 h the medium was supplemented with 10% FCS. The siRNAs were purchase from QIAGEN (QIAGEN, Hilden, Germany). CD44: siCD44_8; RHAMM: siRHAMM_9; LAYN: siLAYN_5.

### Quantitative real-time reverse-transcriptase polymerase chain reaction

Total RNA from cultured cells was isolated from cells with the use of peqGOLD TriFastTM (VWR, Radnor, PA, USA). The lysate was transferred into a 2.0 ml microtube and mixed with 200 µl chloroform, mixed and centrifuged at RT for 15 min at 15,000×*g*. The aqueous phase was transferred into a 1.5 ml microtube and mixed in a ratio of 1:1 with isopropanol p.a. (VWR, Radnor, PA, USA) and centrifuged at 4 °C for 1 h at 21,000×g. The RNA containing pellet was purified with 75% EtOH, centrifuged at 4 °C for 15 min at 21,000×g and subsequently dissolved in RNase free water.

1 µg RNA was transcribed into cDNA with the use of the QuantiTect Reverse Transcription Kit (QIAGEN, Hilden, Germany) according to the manufacturer’s instructions. The cDNA was afterwards diluted in 100 µl RNase free water and the gene expression was investigated with the Platinum SYBR Green qPCR SuperMix-UDG (Invitrogen, Karlsruhe, Germany) using the StepOnePlus RealTime PCRSystem (Thermo Fisher Scientific, Waltham, MA, USA). Primers used are listed in the following table:

**Table:** 

Gene	Primer
18S forward	5′-GCAATTATTCCCCATGAACG-3′
18S reverse	5′-GGCCTCACTAAACCATCCAA-3′
HAS1 forward	5′-TACAACCAGAAGTTCCTGGG-3′
HAS1 reverse	5′-CTGGAGGTGTACTTGGTAGC-3′
HAS2 forward	5′-GTGGATTATGTACAGGTTTGTGA-3′
HAS2 reverse	5′-TCCAACCATGGGATCTTCTT-3′
HAS3v1 forward	5′-CCTTCCCCTACCCAGAGC-3′
HAS3v1 reverse	5′-GAACTGGTAGCCCGTCACAT-3′
HYAL1 forward	5′-CCAAGGAATCATGTCAGGCCATCAA-3′
HYAL1 reverse	5′-CCCACTGGTCACGTTCAGG-3′
HYAL2 forward	5′-TTCACACGACCCACCTACAG-3′
HYAL2 reverse	5′-GTCTCCGTGCTTGTGGTGTA-3′
CD44 forward	5′-GCTATTGAAAGCCTTGCAGAG-3′
CD44 reverse	5′-CGCAGATCGATTTGAATATAACC-3′
RHAMM forward	5′-GAATTTGAGAATTCTAAGCTTG-3′
RHAMM reverse	5′-CCATCATACCCCTCATCTTTGTT-3′
LAYN forward	5′-ATCCTAATCCCCAGCATTCC-3′
LAYN reverse	5′-GGTGTGTTGCTTCTTTGTGC-3′

### Statistical analysis

Datasets with more than two conditions were analyzed with ANOVA plus Tukey’s post hoc test, and datasets with two conditions were analyzed with Student’s *t* test, as appropriate. All statistical analyses were carried out with the software GraphPad Prism 7 (GraphPad Software, La Jolla, CA, USA). Data are presented as the mean ± SEM. Statistical significance was defined as a *p* < 0.05 in the respective test.

## Supplementary Information

Supplementary Information.

## Data Availability

The data presented in this study are available on request from the corresponding author.
