# Supplementary material for: The regulatory effect of hyaluronan on human mesenchymal stem cells’ fate modulates their interaction with cancer cells in vitro
Source: Sci Rep. 2021 Oct 27;11:21229. doi: 10.1038/s41598-021-00754-0 (PMC8551322; doi:10.1038/s41598-021-00754-0)
Supplement: Supplementary file 1 — Supplementary Information. [file 41598_2021_754_MOESM1_ESM.pdf]

Supplementary information

The regulatory effect of hyaluronan on human mesenchymal stem cells' fate modulates their interaction with cancer cells in vitro

Christian Vogeley, Özer Degistirici, Sören Twarock, Jessica Wladarz, Oliver Reiners, Tobias Gorges, Jens W. Fischer, Roland Meisel, Katharina Gorges

This PDF file includes:

Supplementary figures: Figure S1 – S6

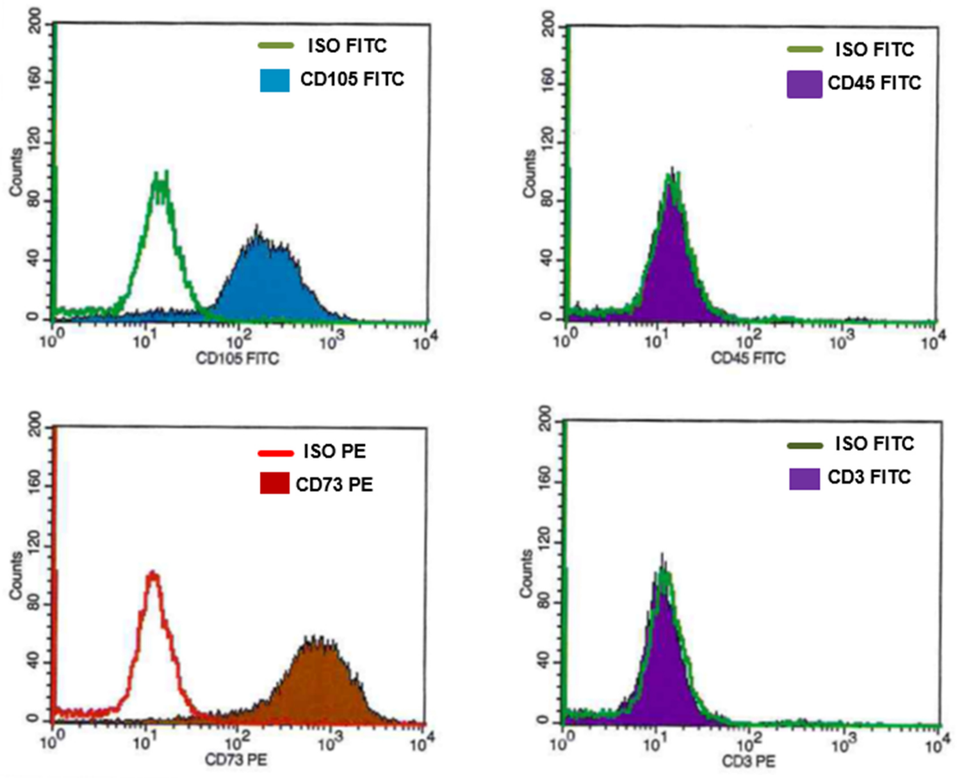

Figure S1: Marker expression profile of islated bmMSCs.Isolated bmMSCs were stained with conjugated antibodies directed against CD73, CD105, CD3 and CD45 and analyzed via flow cytometry. The shown histograms depict the expression of these markers.

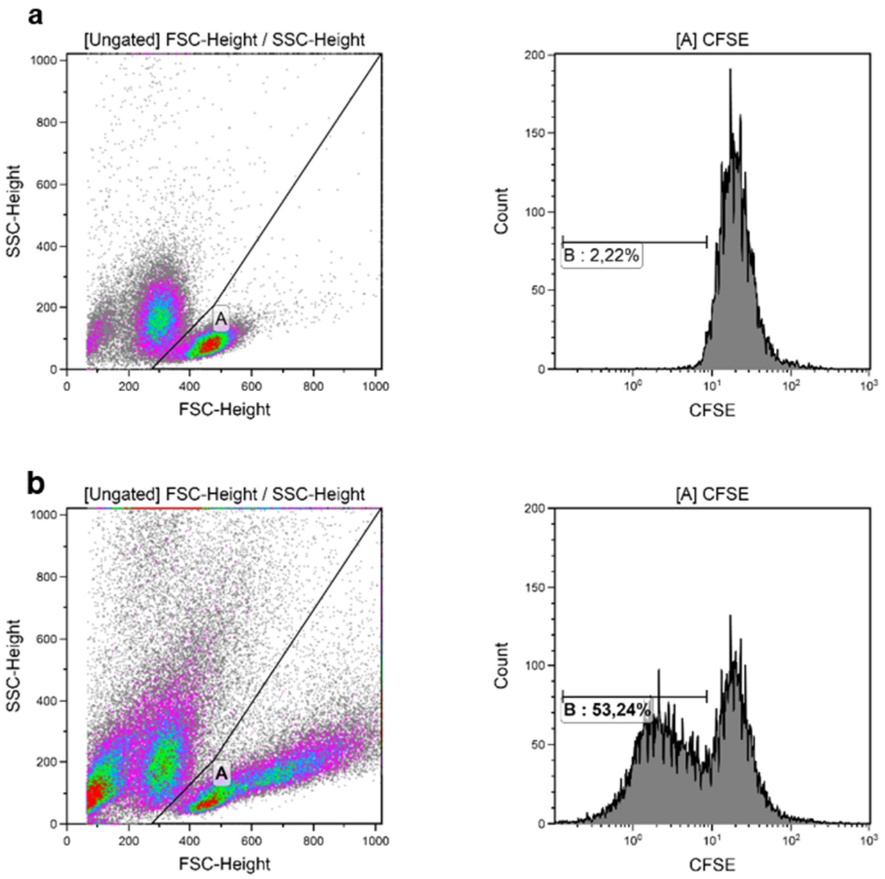

Figure S2: Stimulation of CD3⁺ T-cells with αCD3 and αCD28 antibodies. CD3⁺ T-cells were stained with CFSE and stimulated antibodies directed against CD3 and CD28. Afterwards, the cells were incubated at 37 °C for 6 d. Proliferative rate was determined via flow cytometry. Shown are representative dot plots and histograms of (a) unstimulated and (b) stimulated T-cells.

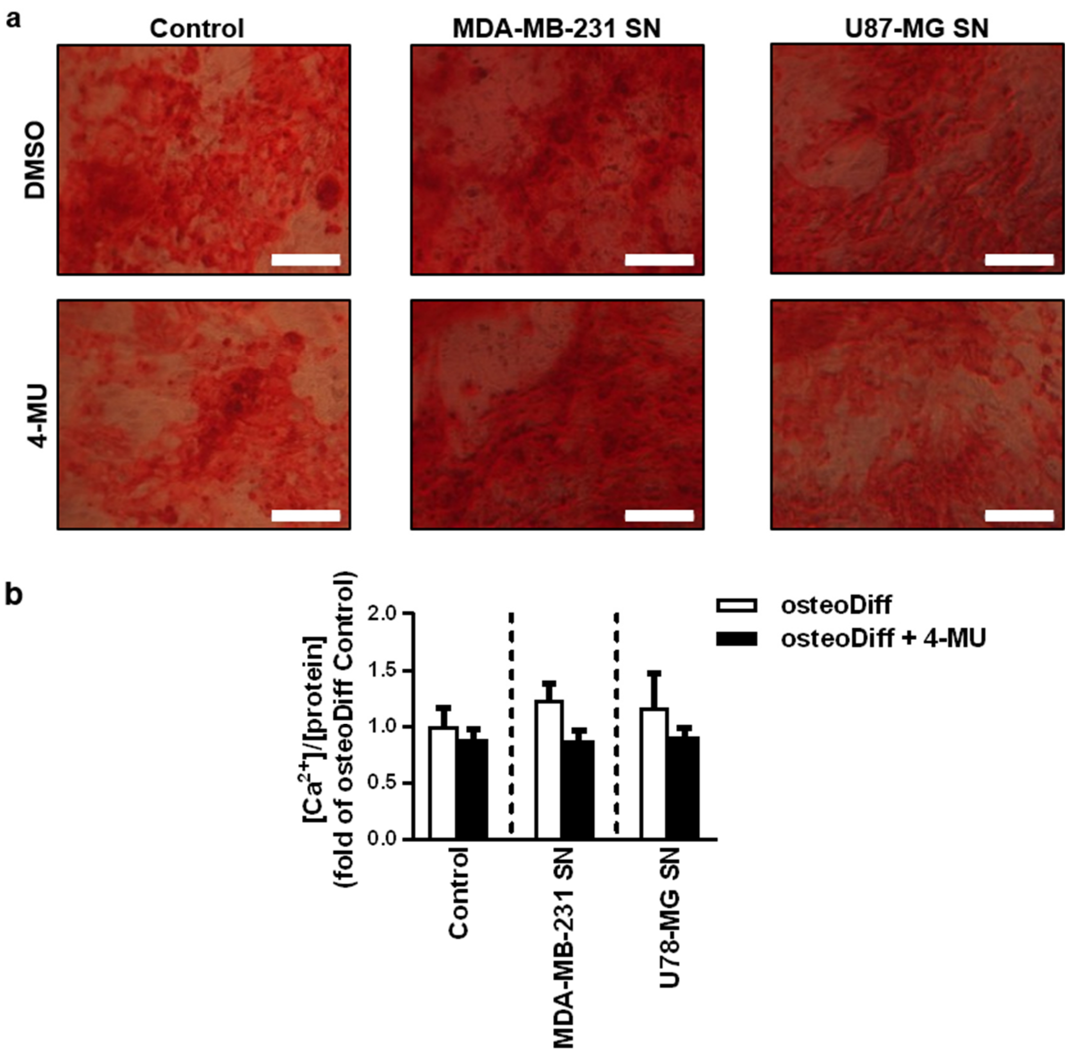

Figure S3: Cancer cell line-derived supernatant does not influence the osteogenic differentiation of mesenchymal stem cells. (a) And (b) BmMSCs were differentiated into osteoblasts with or without 4-MU over a period of 28 d in cancer cell line-derived supernatant or untreated growth medium. (a) Osteogenic differentiation was visualised by staining Ca^{2+}-phosphate depositions on the cell surface with Alizarin S and (b) quantified by measuring the Ca^{2+}-concentration of the cells. The results were normalised to the protein content of the cell lysate. n = 4. Mean ± SEM. *, $p < 0.05$. Scale bar = 200 µm. This figure was prepared with GraphPad Prism (v9.2.0.332, www.graphpad.com).

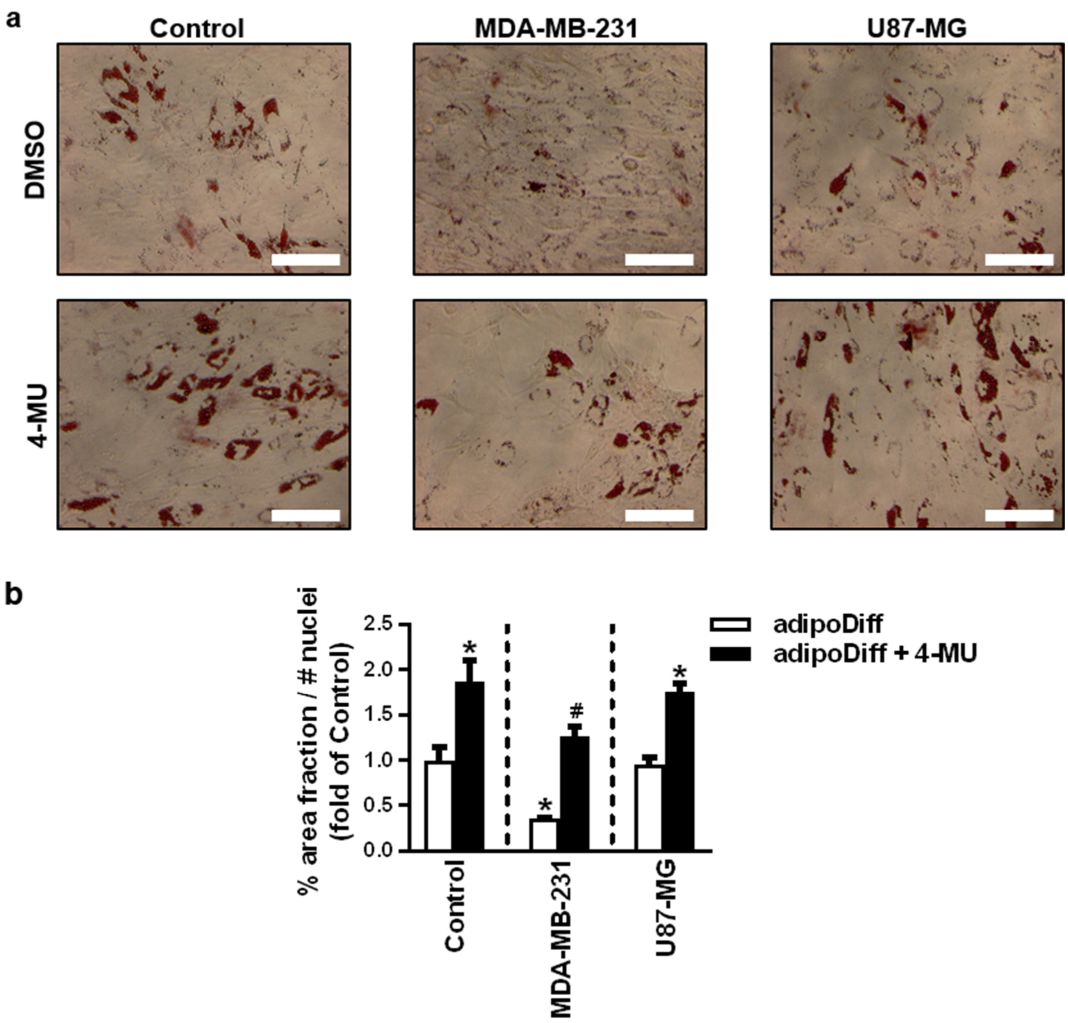

Figure S4: Adipogenic differentiation is inhibited by the invasive breast cancer cell line MDA-MB-231 in a direct coculture. BmMSCs were seeded in a direct co-culture with the cancer cell lines MDA-MB-231 and U87-MG in a ratio of 100:1 and adipogenic stimulated over a period of 28 d. BmMSCs in monoculture were taken as a control. (a) Lipid vesicles were stained with Oil Red O. Scale bar = 200 µm. (b) Adipogenic differentiation was quantified by examining the area fraction of Oil Red O-stained lipid vesicles and normalised to the number of nuclei. *, $p < 0{,}05$ compared to Control/adipoDiff; #, $p < 0.05$ compared to MDA-MB-231/adipoDiff. n = 4. Mean ± SEM. This figure was prepared with GraphPad Prism (v9.2.0.332, www.graphpad.com).

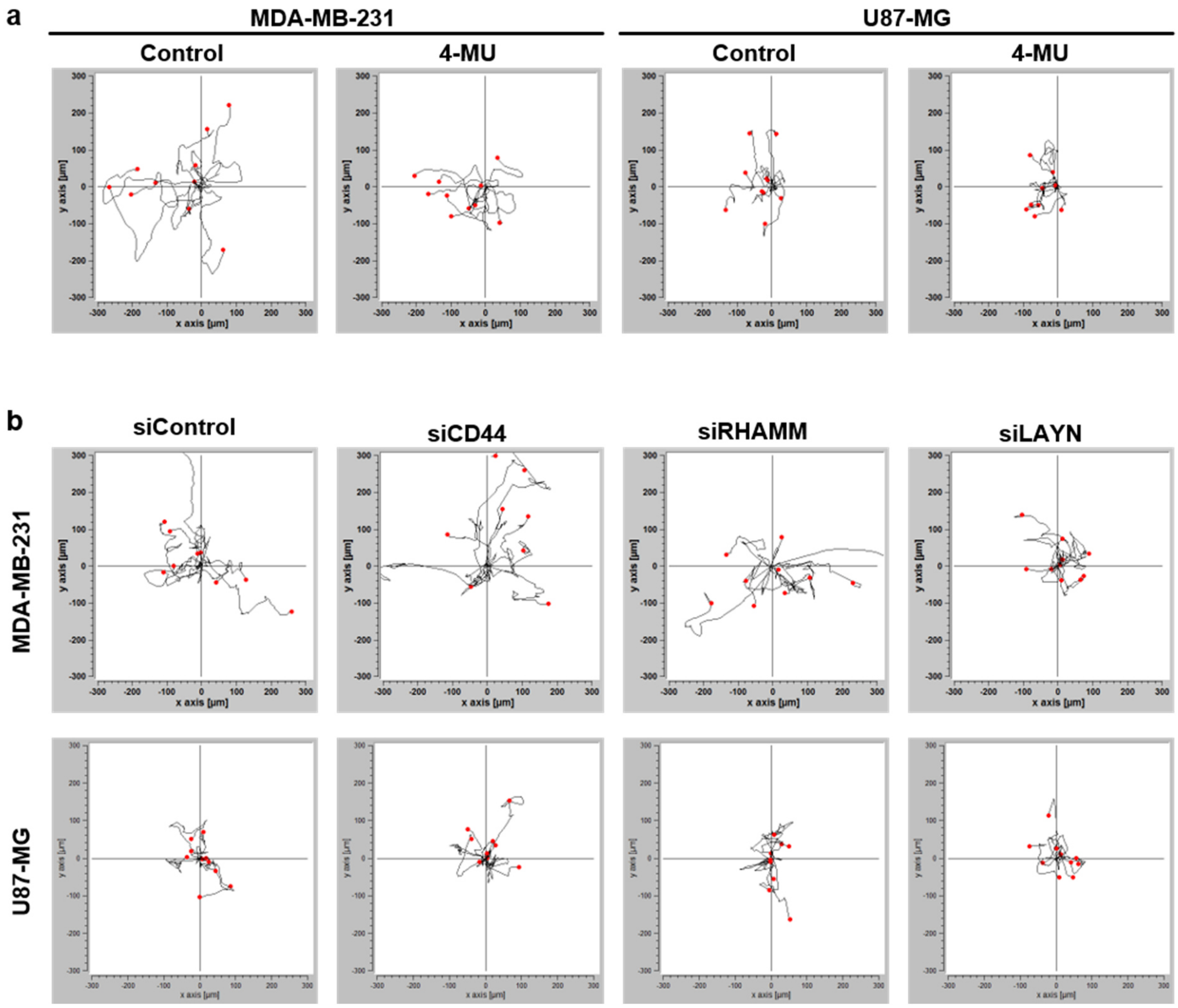

Figure S5: Depletion of the hyaluronan system reduces the interaction between invasive breast cancer cells and mesenchymal stem cells. (a) BmMSCs were treated with 4-MU over a period of 72 h. 4-MU treated bmMSCs were then seeded in co-culture with MDA-MB-231 and U87-MG cells. Shown is the accumulated distance of cancer cells in juxtaposition with bmMSCs after 24 h. (b) The expression of HA interacting receptors CD44, RHAMM and LAYILIN was depleted in the cancer cells MDA-MB-231 and U87-MG and co-cultured with bmMSCs. Shown is the accumulated distance of cancer cells in juxtaposition with bmMSCs after 24 h. The origin of the coordinate system depicts the starting point of and the red dot the endpoint of analysed cells. n = 4.

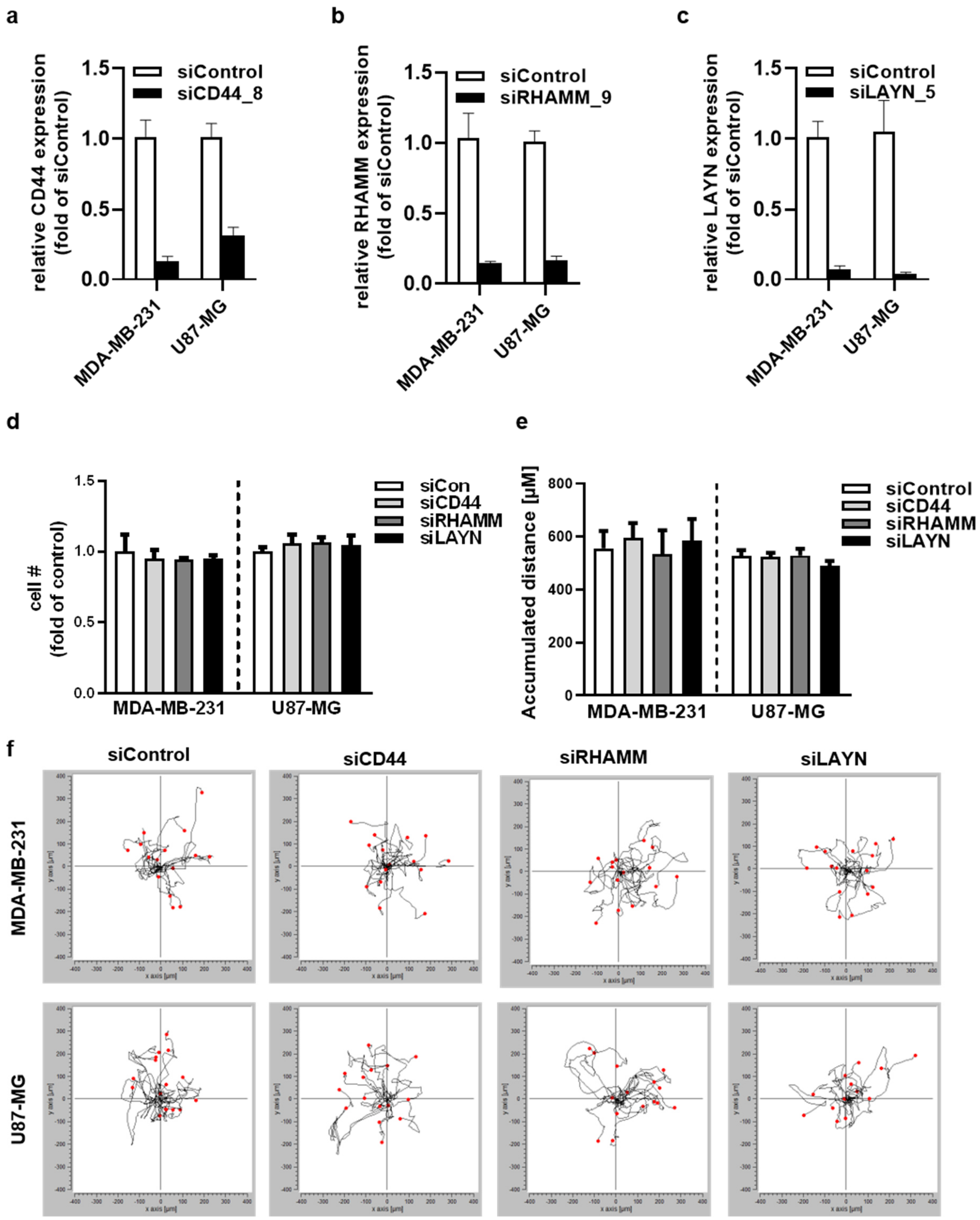

Figure S6: Effect of HA receptor knockdown on the cell number and motility of MDA-MB-231 and U87-MG cells in monoculture. The expression of the genes for the HA interacting receptors CD44, RHAMM and LAYN was suppressed via siRNA. The efficiency of the knock downs of (a) CD44, (b) RHAMM and (c) LAYN was validated via qRT-PCR analysis. (d) Cell number was determined by counting the cancer cells with a Neubauer chamber. (e) The accumulated distance was measured via time-lapse microscopy over a period of 24 h and normalised to the respective control. (e) Coordinate systems show the track of single analysed cells, whereby the origin depicts the starting point and the red dot the endpoint of each cell. n = 4. Mean ± SEM. (f) Shown is the accumulated distance of MDA-MB-231 and U87-MG cells after control treatment or knock down of CD44, RHAMM or LAYN after 24 h. The origin of the coordinate system depicts the starting point of and the red dot the endpoint of analysed cells. n = 4. This figure was prepared with GraphPad Prism (v9.2.0.332, www.graphpad.com).
